# Self-views and aggression in boys referred for disruptive behavior problems: self-esteem, narcissism, and their interaction

**DOI:** 10.1007/s00787-019-01347-z

**Published:** 2019-05-31

**Authors:** Wieteke Hiemstra, Esmée E. Verhulp, Sander Thomaes, Bram Orobio de Castro

**Affiliations:** Department of Psychology, Utrecht University, P.O. Box 80.140, 3508 TC Utrecht, The Netherlands

**Keywords:** Aggression, Self-views, Narcissism, Self-esteem, Conduct problems, Psychopathology

## Abstract

How do children with aggressive behavior problems view themselves? The present research seeks to answer this question by examining the self-views (i.e., self-esteem and narcissism) of boys referred for disruptive behavior problems. In Study 1 (*N *= 85, Mage= 10.8 years), we examined relations between self-views and self-reported and parent-reported aggression; in Study 2 (*N *= 73, Mage= 11.8 years), we examined relations between self-views and teacher-reported aggression. We found narcissism to be related with self-reported aggression, but not with parent- and teacher-rated aggression. Children with narcissistic traits were more aggressive according to themselves, and these links were independent of children’s level of self-esteem. Self-esteem was not significantly associated with aggression according to children themselves, their parents, nor their teachers. We encourage scholars to explore the possibility that interventions that target characteristics of narcissistic self-views (e.g., perceived superiority, sensitivity to negative feedback) can effectively reduce aggressive behavior in boys referred for behavior problems.

## Introduction

How do children with aggressive behavior problems view themselves? Do they typically feel bad about themselves, and perhaps act out against others to cope with their self-doubt? Or, alternatively, are they more likely to have inflated, grandiose self-views that instigate them to aggress when experiencing ego-threat?

It has long been assumed that children with aggression problems are prone to suffer from negative self-views. Accordingly, attempts to reduce children’s aggression problems often seek to enhance children’s level of self-esteem [e.g., 1–4]. The literature on typically developing children, however, suggests that aggression may also stem from “threatened egotism”—inflated, narcissistic self-views that are jeopardized, rather than from low self-esteem per se [5–8]. One might be tempted to infer, then, that intervention-techniques that seek to boost children’s self-esteem in order to reduce aggression are ill-advised. Unfortunately, however, research on the self-views of children referred for aggression problems—those for whom intervention is particularly urgent—is scarce. Findings obtained in population samples of youth do not necessarily generalize to at risk or clinical samples: there are important differences in both individual (i.e., temperament, social cognition) and contextual (i.e., family and peer interaction) determinants and correlates of aggressive behavior in these groups [e.g., 9–11]. The present research seeks to contribute to filling this lacuna by examining the self-views (i.e., self-esteem and narcissistic traits) of boys referred to special education for their disruptive behavior problems.

## Self-esteem and aggression

Self-esteem refers to children’s global feelings of self-worth [12, 13]. There are good reasons why it was long assumed that aggressive youth have low self-esteem. For example, low self-esteem may weaken ties to society, potentially leading to nonconformity to social norms [13]. Also, children may be likely to protect themselves against negative self-feelings by externalizing blame for their own perceived shortcomings [14, 15]. However, the link between self-esteem and aggression is empirically controversial. In population samples of youth, some studies found evidence for a small link between low self-esteem and aggression [e.g., 16–18], but other research has suggested that such links may be at least partially accounted for by third variables (e.g., family characteristics such as parental education level) associated with both self-esteem and aggression, and thus, that it is not low self-esteem per se driving the effects on aggression [19]. Still other research found no link between low self-esteem and aggression at all [e.g., 5, 20, 8], or found that inflated, unrealistically positive (rather than deflated) self-views are linked to aggressive behavior [7, 21]. Thus, in population-based samples, there is no evidence for a substantial or robust link between low self-esteem and aggressive behavior.

From a clinical perspective, research in population samples is informative, but it is even more important to know how children with the most serious aggression problems—i.e., those who receive treatment—typically view themselves. There are reasons to anticipate that links between self-esteem and aggression may differ in clinical samples as compared to population samples. Children with disruptive behavior problems are more likely to exhibit vulnerabilities that may contribute to low self-esteem (e.g., low IQ, difficult temperament, [9]). They also tend to have more prevalent histories of aversive social experiences, such as coercive family interactions, peer rejection, or neglect [e.g., 22, 10, 23, 11]. These factors may well impact the link between children’s self-views and their aggressive behavior. For example, parental neglect or lack of warmth is known to both undermine healthy self-esteem development and cultivate children’s aggressive behavior [24, 25], suggesting that low self-esteem may be a more potent risk factor for aggressive behavior in clinical samples of youth.

However, some children with disruptive behavior problems may have vulnerabilities in the processing of social information, which may impair their accommodation of negative social feedback. These vulnerabilities may be associated with co-occurring attention-deficit/hyperactivity disorder (ADHD) and autism spectrum disorder (ASD). Indeed, some evidence suggests that boys with co-occurring ADHD and aggression problems tend to display positive illusory bias, and overestimate their own competences [26–28]. The aggression problems of these children thus seem unlikely to be linked to negative self-views. Furthermore, subsets of children with ASD display aggression [30], and often show deviant self-view development due to difficulties in incorporating social feedback into their self-views [29]. Thus, a potential link between low self-esteem and aggressive behavior among children with disruptive behavior problems might be moderated by co-morbid developmental disorders.

Surprisingly, despite its clinical relevance, research into the self-esteem–aggression link in clinical samples of children, is scarce. Findings from studies with adolescents who dropped out of school for various academic, internalizing, or externalizing problems, tentatively suggest that a relation between low self-esteem and aggressive behavior may exist among adolescents with such diverse problems [31]. However, whether these findings in adolescents with heterogeneous problems can be generalized to children with severe disruptive behavior problems is not clear. One study which included a clinical sample of youth with oppositional defiant disorder found a link between low self-esteem and conduct problems [59]. However, no other developmental disorders were included. Thus, even though enhancing self-esteem is a common goal for current interventions to reduce severe aggressive behavior problems, the scientific evidence on the self-views of children showing such problems is limited. We sought to address this relative lack of evidence. The first aim of this study was to test whether self-esteem and aggressive behavior are negatively related among children with disruptive behavior problems, and whether this relation is moderated by co-morbid developmental disorders.

## Narcissism and aggression

It has been argued that other qualities of self-views than self-esteem may be relevant to aggressive behavior. Narcissism refers to a sense of grandiosity and entitlement, and a strong need to be seen and admired by others [32, 33]. Although extreme levels of narcissism may reflect pathology, narcissism is more typically conceived of as a trait on which children in the general population vary. One way in which trait narcissism differs from self-esteem, is that it involves unrealistically positive self-views and a strong sense of superiority, with a need for validation of these feelings by others [34–36]. Narcissism has long been thought to predispose individuals to aggress, because when narcissists’ fragile positive self-views are threatened (e.g., when they are criticized, rejected, or ridiculed) they tend to lash out aggressively, perhaps in an attempt to protect their ego [6, 8, 37]. Studies have indeed shown consistent links between narcissism and various forms of aggressive behavior in population samples of children and adolescents [e.g., 20, 38–40, 8].

Just as for low self-esteem, relations between narcissism and aggressive behavior have primarily been studied in population samples, even though there is ample reason to anticipate this link to differ in clinical samples. Children referred for behavior problems often have deviant social histories [9, 22], and may be exposed to parental and peer modeling of aggressive behavior as a means to ward off negative (self) evaluation, ‘save face’ or ‘command respect’ [41]. Studies conducted in samples of adolescents at risk for a variety of academic, internalizing and externalizing problems suggest that narcissism is indeed related to (proactive) aggressive behavior [31, 42]. Also, one study that included a clinical sample of youth with oppositional defiant disorder found a positive relation between narcissism and conduct problems [58]. However, no other developmental disorders were included. Perhaps surprisingly, though, no other studies analyzed this relation among children referred, specifically, for disruptive behavior problems. The second aim of our study was to examine whether narcissism and aggressive behavior are related among boys with disruptive behavior problems in middle childhood.

Importantly, self-esteem and narcissism are not just two sides of the same coin—they show only weak-to-moderate overlap [24, 32]. For example, narcissism differs from self-esteem in its characteristic cognitions (i.e., perceived superiority versus satisfaction with oneself) and origins (i.e., parental overvaluation versus parental warmth [34]). In fact, theory and research suggest that there might be different subtypes of narcissists, who differ in their level of self-esteem [e.g., 8, 20, 39], suggesting that self-esteem might interact with narcissism in predicting aggression. It is interesting to explore, then, if and how interactions between self-esteem and narcissism are associated with aggression among children with disruptive behavior problems. The third and final aim of our study was to examine this interaction.

## The present research

The present research is the first to investigate the links between self-esteem, narcissism, and aggression in clinical samples of boys referred to special education for severe disruptive behavior problems. Given that parent, teacher and child reports of aggression tend to correlate only modestly [e.g., 58], we decided to use a multi-rater approach. In Study 1, we examined self-reported and parent-reported aggression as outcome measures; in Study 2, we examined teacher-reported aggression as an outcome measure. In both studies, we limited the samples to boys, because very few girls are referred to special education for severe disruptive behavior problems, and the etiology of their behavior problems seems to be markedly different [43].

In both studies, we hypothesized that narcissism would be positively related and self-esteem would not be related with aggression. We expected the relation between narcissism and aggression to be stronger among children with higher self-esteem [8]. Moreover, in both studies we explored whether the links between self-views and aggression differ for two prevalent subgroups of clinically referred children: those who show co-occurring autism spectrum disorder (ASD), or another behavioral disorder [e.g., oppositional defiant disorder (ODD), and ADHD].

## Study 1

### Methods

#### Participants

Participants were 85 Dutch boys aged 8–13 (mean age = 10 years, 8 months). They were recruited from seven schools providing special elementary education for children with behavior problems. These schools were randomly selected for participation from all schools providing this type of special education. In the Netherlands, children are exclusively referred to this form of special education if the severity of their behavior problems significantly impairs social functioning and prohibits participation in regular education, as judged by parents, teachers, and diagnosticians. About 2.5% of the school-aged population is referred to this type of education [44]. Participating schools were situated in rural and suburban areas in the center and south of the Netherlands, but boys came from across the country to attend these schools. Participants were diagnosed with a DSM-IV disorder by multidisciplinary teams of professionals (obtained from school care records; 30.6% ADHD only, 8.2% ODD or CD only, 7.1% both ADHD and ODD or CD, 25.9% ASD, 20% ADHD and ASD, 8.2% other).

#### Measures

*Instrument for reactive and proactive aggression* We measured aggressive behavior using the Instrument for Reactive and Proactive Aggression (IRPA [45]). Both children and parents completed this measure. The IRPA consists of seven “form” items (e.g., “How often did the child/you hit someone in the past 6 months?”), and six “function” items (e.g., “How often did he do this because he was angry?”). For this study, only form-items were used. Items are rated along a 5-point Likert scale ranging from 0 (never) to 4 (multiple times). Items were averaged to obtain a total aggression score. Good discriminant, convergent, and construct validity have been demonstrated for the IRPA [45]. In the current sample, alpha for child-report and parent-report aggression was good (Cronbach’s *α* = .79 and .82, respectively).

*Childhood narcissism scale* We measured narcissism using the Childhood Narcissism Scale (CNS), a one-dimensional self-report measure of relatively stable individual differences in childhood narcissism [46]. Ten items (e.g., “I am a great example for other kids to follow”) are rated along a 4-point Likert scale ranging from 0 (not at all true) to 3 (completely true). Responses were averaged across items, reliability in the current sample was good (Cronbach’s *α* =.81).

*Self-perception profile for children* We measured self-esteem using the Global Self-Worth subscale of the Self-Perception Profile for Children [47]. Children rated six items (e.g., “Some kids are happy with themselves as a person”) along a 4-point Likert scale ranging from 0 (I am not like these kids at all) to 3 (I am exactly like these kids). Responses were averaged across items, with acceptable reliability (Cronbach’s *α* = .67).

#### Procedure

Schools distributed parental consent forms. Only boys who received active consent from their parents/caregivers were included in the study. We asked schools to distribute the consent forms among 250 children. 96 children got active consent to take part in the study (participation rate = 38%), 85 of them were boys. Diagnostic information was retrieved from school care records. Children completed questionnaires individually under the supervision of a trained research assistant (i.e., a female psychology student). After completing the questionnaire, we gave children stickers to thank them for their voluntary participation.

#### Missing values

Eight parents returned incomplete or no IRPA questionnaires. Because these data were missing completely at random [Little’s MCAR test: *χ*^2^(3) = 3.75*, p *= .29], data from these parents were imputed using multiple imputation in SPSS. Child- and parent-reported aggression, narcissism, self-esteem and the interaction term were included as predictors in the imputation model. We generated 20 datasets to impute the missing values.

### Results

Table 1 shows the correlations between the main variables in Study 1. Aggression reported by children and their parents was positively correlated. Participants with an ASD diagnosis did not differ significantly from participants without ASD diagnosis in self-esteem and narcissism (*p *=.68 and *p *= .58, respectively).

**Table 1:** Descriptive statistics and correlations

	Mean	SD	Aggression parent	Aggression child	Narcissism
Aggression parent	.98	.66	–	–	–
Aggression child	1.00	.63	.38**	–	–
Narcissism	1.40	.62	.20	.29**	–
Self-esteem level	2.22	.49	− .10	− .18	.01

Aggression parent is child aggression rated by parents

Aggression child is self-reported child aggression

**p < .01

We used regression analyses to examine the association between narcissism and aggression, and the hypothesized moderating role of self-esteem. The two aggression measures served as the dependent variables, so we carried out two separate analyses for the two raters. Narcissism and Self-Esteem were centered and entered in Step 1, and their interaction in Step 2. For Parent-Reported Aggression (see Table 2), no main- or interaction effects were found. For Child-Reported Aggression (see Table 3), a positive main effect of Narcissism on Aggression was found; as predicted, more narcissistic boys were more aggressive. No significant main effect of self-esteem was found. The interaction between narcissism and self-esteem was also not significant.

**Table 2:** Summary of regression of aggression as reported by parents on narcissism and self-esteem (*N* = 85)

Model/variables	*R*^2^	*B*	SE *B*	*β*
Model 1	.05			
Narcissism		.13	.07	.20
Self-esteem		− .07	.07	− .11
Model 2	.06			
Narcissism		.14	.08	.21
Self-esteem		− .07	.08	− .10
Narcissism* self-esteem		− .05	.09	− .07

*p < .05; **p < .01

**Table 3:** Summary of regression of aggression as reported by children on narcissism and self-esteem (*N* = 85)

Model/variables	*R*^*2*^	*B*	SE *B*	*β*
Model 1	.12**			
Narcissism		.29	.11	.29**
Self-esteem		− .24	.13	− .19
Model 2	.14*			
Narcissism		.27	.11	.27**
Self-esteem		− .26	.13	− .20
Narcissism* self-esteem		.41	.26	.16

*p < .05; **p < .01

We further explored whether the link between narcissism and aggression would be moderated by ASD diagnosis for the child-reported aggression. We performed an additional regression analysis which included an interaction term with ASD. No significant main or interaction effects involving ASD were found (*β* = − .13, *p* = .20, and *β *= − .20, *p* = .06, respectively).

### Discussion

In Study 1, we examined the associations between self-views and aggression for a clinically referred group of boys with disruptive behavior problems. Boys who considered themselves more aggressive showed higher levels of narcissism, but not lower levels of self-esteem. However, boys who were considered by their parents to be more aggressive were not more narcissistic, nor did they have lower self-esteem. Also, we found no evidence that the link between narcissism and self-reported aggression was contingent upon children’s level of self-esteem, or diagnosis with ASD.

Our findings are partly consistent with previous research in general population samples of youth, showing that narcissism can predispose children to be aggressive [e.g., 20, 38–40, 8]. The absence of a significant moderating role of self-esteem, however, was unanticipated, given that clinical theory and research suggest that different subtypes of narcissists (characterized by differential levels of self-esteem) may show different levels of aggression [e.g., 8, 20, 39].

One possible explanation for the absence of this interaction is that narcissistic aggression is mainly rooted in fluctuating self-esteem (e.g., triggered by feelings of threat or shame), rather than low self-esteem per se. In Study 2, we therefore included an index of self-esteem stability as a potential correlate of aggression and moderator of the narcissism–aggression link.

## Study 2

The goal of Study 2 was to obtain a more comprehensive understanding of the links between self-views and aggression among clinically referred children, by (1) examining associations between children’s self-views and teacher-reported aggression, and (2) examining stability of self-esteem as an additional potential correlate and moderator of aggression.

Self-esteem stability refers to the magnitude of short-term fluctuations in immediate feelings of self-worth [48]. Self-esteem instability is a trait-like characteristic that is (negatively) associated with, but distinct from, level of self-esteem [49]. Self-esteem stability has been considered a proxy for the vulnerability of children’s self-views. Studies in adults have shown that individuals with high yet unstable self-esteem tend to be more hostile compared to those with (stable or unstable) low self-esteem [48, 50]. Furthermore, research in adults has found some evidence that self-esteem stability is a unique predictor of aggression [51]. We therefore hypothesized that self-esteem stability would be a unique predictor of aggression, and would also moderate the link between narcissism and aggression.

### Methods

#### Participants

The sample consisted of 73 Dutch boys aged 8–13, with a mean age of 11 years and 8 months. These boys were recruited from four schools providing special elementary education for children with disruptive behavior problems (i.e., the same school type as in Study 1), located in rural and suburban regions in the Netherlands. The schools were different from those taking part in Study 1. Participants were diagnosed with a DSM-IV disorder by multidisciplinary teams of professionals (obtained from their school care records; 17.8% ADHD only, 8.2% ODD or CD only, 8.2% both ADHD and ODD or CD, 27.4% ASD, 17.8% ADHD and ASD, 20.6% other).

#### Measures

As in Study 1, participants completed the Childhood Narcissism Scale ([46]; Cronbach’s *α* =.71), and the Self-Perception Scale for Children ([47]; Cronbach’s *α *= .82). For self-esteem stability, we used a slightly adapted version of this latter measure, by asking children to report on a day-to-day basis, for 5 consecutive days, how they feel “right now”. We found acceptable-to-good reliability for the daily self-esteem assessments (*α*s ranging between .67 and .82). Following standard procedures [e.g., 50] self-esteem stability was calculated for each participant as the within person standard deviation of his individual state self-esteem scores across the 5 weekdays. This means that higher scores on this measure mean more instability in self-esteem for that specific child. Teachers completed the Instrument for Reactive and Proactive Aggression (IRPA; [45]; Cronbach’s *α* = .92).

#### Procedure

We sent out parental consent forms via the schools. Only boys who received active consent from their parents/caregivers were included in the study (consent rate = 60%). Diagnostic information was retrieved from school care records. This study was part of a larger intervention study. Participants completed daily assessments of self-esteem in individual testing sessions at the school grounds. The other questionnaires were completed 1 week earlier in class. Participants received a small present (i.e., a pen or eraser) at the third and the fifth day to thank them for voluntary participation. We obtained ethics approval from our local ethics review board at Utrecht University, Faculty of Social and Behavioural Sciences.

#### Missing values

For 14 boys, one or multiple questionnaires were missing, and they were therefore excluded from the study. Because most of them (10) failed to complete multiple questionnaires, multiple imputation was not a viable strategy to impute their scores. The questionnaires were missing completely at random [Little’s MCAR test: *χ*^2^(7) = 7.71, *p *= .36], because of illness or therapy-related school absence.

### Results

Table 4 shows the correlations between the main Study 2 variables. No significant associations were found between any of the self-view measures and teacher-reported aggression. Self-esteem level and self-esteem stability were significantly negatively correlated; participants with lower self-esteem showed more self-esteem instability. Again, no significant differences were found on any of the self-view variables for participants with and without ASD diagnosis.

**Table 4:** Descriptive statistics and correlations

	Mean	SD	Aggression teacher	Narcissism	S-E level
Aggression teacher	.91	.88	–	–	–
Narcissism	1.37	.53	− .14	–	–
S-E level	2.24	.71	− .18	− .03	–
S-E stability	.30	.28	.04	.08	− .33**

Aggression teacher is child aggression rated by teachers

*S-E* is self-esteem

**p < .01

To examine the links between narcissism and teacher-reported aggression and the moderating role of self-esteem level and stability, we performed two separate regression analyses. As in Study 1, we regressed Aggression on the centered variables Narcissism, Self-Esteem or Self-Esteem Stability to analyze any main effects in Step 1, and added the Self-Esteem Level and Stability interaction-terms with Narcissism to these models in Step 2.

We found no significant main effects for Narcissism or Self-Esteem Level, nor did we find an interaction between these variables (see Table 5). Again, we explored whether having an ASD diagnosis would influence the findings by adding ASD and its interaction with Narcissism to the regression equation (45% of the participants were diagnosed with ASD). A main effect was found (*β *=− .32, *p *< .01), indicating that teachers reported higher levels of aggression for children without an ASD diagnosis. The interaction was non-significant (*β *=.07, *p *= .57).

**Table 5:** Summary of regression of aggression reported by teachers on narcissism and self-esteem level (*N* = 73)

Model/variables	*R*^2^	*B*	SE *B*	*β*
Model 1	.06			
Narcissism		− .25	.19	− .15
Self-esteem		− .24	.15	− .19
Model 2	.06			
Narcissism		− .15	.19	− .15
Self-esteem		− .24	.15	− .19
Narcissism* self-esteem		.02	.27	.01

*p < .05; **p < .01

In the second analysis, the main and interaction effects of Self-Esteem Stability were not significant (Table 6) and no interaction between ASD and Narcissism was found (*β *=.01, *p *= .94).

**Table 6:** Summary of regression of aggression reported by teachers on narcissism and self-esteem variability (*N* = 73)

Model/variables	*R*^2^	*B*	SE *B*	*β*
Model 1	.02			
Narcissism		− .24	.19	− .15
Self-esteem stability		.15	.37	.05
Model 2	.02			
Narcissism		− .25	.20	− .15
Self-esteem stability		.18	.40	.06
Narcissism* self-esteem stability		− .15	.83	− .02

*p < .05; **p < .01

## General discussion

This research was the first to examine the links between multiple aspects of children’s self-views and aggression in boys referred for disruptive behavior problems. We used a multi-method approach to study the links between narcissism, self-esteem and aggression in two independent samples. Consistent with previous findings in population-based samples of youth [e.g., 20, 38, 39], narcissistic children self-reported to be more aggressive. However, according to their parents and teachers, narcissistic children were not more aggressive. As predicted, regardless of informant, self-esteem was not associated with aggression [e.g., 5, 20, 8].

One explanation for our finding that narcissism is unrelated to parent- and teacher-reported aggression may be found in the context in which the children’s aggression takes place. Narcissistic aggression is typically triggered in situations in which children experience ego-threat (i.e., interpersonal contexts [e.g., 6]). Such aggression may be less likely to occur in the settings in which special education teachers usually observe their students: Highly structured and regulated, small group settings. Also, parents see their children outside of the classroom, but are not present in all situations where children play with others. In addition, it is possible that the association between narcissism and aggression we found in Study 1 was partly due to biased self-reports of aggression. In theory, this correlation may therefore have been due to children high on narcissism also over-reporting their own aggressive behavior. Given the social undesirability of aggressive behaviors, this may not seem a likely reporting bias for children high on narcissism, but we cannot rule out the possibility that some of these children consider aggressive behavior a positive characteristic they may be tempted to exaggerate [41].

As a first step to furthering our understanding of the narcissism–aggression link among referred youth, we took the opportunity to explore whether this link may differ for those with and without ASD diagnosis. We found no evidence for such moderation, but note that statistical power did not allow for identifying small effects. Further research is therefore warranted.

Low self-esteem was not significantly related to any aggression measure in our studies. Thus, our findings lend no support for the hypothesis that lower self-esteem may instigate aggressive behavior among children with behavior problems. It could be argued that our sample sizes were modest, which is true. Nonetheless, the range of non-significant correlations does suggest that even if a link between low self-esteem and aggression would exist among these boys, it would be small (explained variance *r*^2^ of 1–4%), and perhaps too small to warrant low self-esteem as a main focus of treatment for these boys. On the other hand, we cannot rule out the possibility that this relation was absent in the present sample for the very reason that participants may have already participated in (educational or parental) self-esteem enhancement (that did not decrease their behavior problems sufficiently) before participating in the present study. Similarly, we cannot be sure that earlier intervention has not inadvertently promoted narcissism through overestimation [24].

This research has limitations. Although we used a multi-method approach, we relied on reported rather than behavioral measures of aggression. Observing the link between self-views and aggression in an in vivo setting [e.g., 8] would be a valuable complement to this work, tapping the interpersonal context in which narcissistic aggression may be most apparent. Such experimental research could also address causality, which was not possible with our cross-sectional design. Given the ethical implications of conducting such experiments with children with disruptive behavior problems, we opted to first conduct the present, less invasive study. The present research may provide the groundwork for future experimental or intervention studies. For example, by experimentally studying the effects of procedures to (temporarily) reduce narcissistic vulnerability on aggressive behavior.

This research also has several strengths. First, it is the first to comprehensively test the links between self-views and aggression in samples of referred youth. Second, we used multiple informants to test our hypotheses. Aggression is highly context-dependent, and so the inclusion of multiple informants helps to validly assess individual differences in aggression and inform intervention possibilities.

Future research should further explore the links between self-views and aggression in clinically referred samples of youth, preferably using experimental and longitudinal designs. Because clinical populations tend to be heterogeneous, differential developmental trajectories should be explored. For example, whereas deviant self-views may be an antecedent of aggression for some children, they may be a consequence of children’s aggressive predispositions as well. Furthermore, there are different forms and functions in aggression [45, 53]. Some narcissists may use more proactive, instrumental aggression (e.g., because they feel they can gain status from behaving aggressively [54]), others might reactively aggress because they see others as being hostile and unjust [e.g., 55].

Research has shown that children with positive views of themselves but negative views of their peers (“I’m OK but you’re not”) are especially aggressive [56]. It may be that narcissistic children, who typically hold such discrepant self- and peer-views, consider their aggression as a justified response to perceived hostility. This would be consistent with experimental research on the mediating role of hostile intent attribution in the link between narcissism and aggression [52]. This would suggest that rather than boosting children’s level of self-esteem (perhaps unwittingly contributing to the “I’m OK” part of the aggression equation), interventions would better focus on helping children adopt a more realistic and accepting view of one’s strengths and weaknesses [32, 57].

Our research challenges the notion that boosting aggressive children’s level of self-esteem should be a common aim for intervention. Indeed, even among referred children, there is little evidence that aggressive children primarily suffer from low self-esteem. We encourage scholars, instead, to explore the possibility that interventions that target narcissistic self-views or its associated characteristics (e.g., perceived superiority, sensitivity to negative feedback) might effectively reduce aggression in subsets of referred boys.
